# Comprehensive Annotation and Expression Profiling of C2H2 Zinc Finger Transcription Factors across Chicken Tissues

**DOI:** 10.3390/ijms251910525

**Published:** 2024-09-30

**Authors:** Shuai Chen, Jiayao Jiang, Wenxiu Liang, Yuchen Tang, Renzhe Lyu, Yun Hu, Demin Cai, Xugang Luo, Mingan Sun

**Affiliations:** 1 Institute of Comparative Medicine, College of Veterinary Medicine, Yangzhou University, Yangzhou 225009, China; chenshuai@yzu.edu.cn (S.C.); dx120220184@stu.yzu.edu.cn (J.J.); liangwenxiu2021@163.com (W.L.); mx120231060@stu.yzu.edu.cn (Y.T.); mx120221021@stu.yzu.edu.cn (R.L.); 2 College of Animal Science and Technology, Yangzhou University, Yangzhou 225009, China; huyun@yzu.edu.cn (Y.H.); demincai@yzu.edu.cn (D.C.); 3 Joint International Research Laboratory of Important Animal Infectious Diseases and Zoonoses of Jiangsu Higher Education Institutions, Yangzhou University, Yangzhou 225009, China; 4 Jiangsu Co-Innovation Center for Prevention and Control of Important Animal Infectious Diseases and Zoonosis, Yangzhou University, Yangzhou 225009, China; 5 Joint International Research Laboratory of Agriculture and Agri-Product Safety of Ministry of Education of China, Yangzhou University, Yangzhou 225009, China

**Keywords:** chicken, transcription factor, zinc finger protein, KRAB, SCAN, expression

## Abstract

As the most abundant class of transcription factors in eukaryotes, C2H2-type zinc finger proteins (C2H2-ZFPs) play critical roles in various biological processes. Despite being extensively studied in mammals, C2H2-ZFPs remain poorly characterized in birds. Recent accumulation of multi-omics data for chicken enables the genome-wide investigation of C2H2-ZFPs in birds. The purpose of this study is to reveal the genomic occurrence and evolutionary signature of chicken C2H2-ZFPs, and further depict their expression profiles across diverse chicken tissues. Here, we annotated 301 C2H2-ZFPs in chicken genome, which are associated with different effector domains, including KRAB, BTB, HOMEO, PHD, SCAN, and SET. Among them, most KRAB-ZFPs lack orthologues in mammals and tend to form clusters by duplication, supporting their fast evolution in chicken. We also annotated a unique and previously unidentified SCAN-ZFP, which is lineage-specific and highly expressed in ovary and testis. By integrating 101 RNA-seq datasets for 32 tissues, we found that most C2H2-ZFPs have tissue-specific expression. Particularly, 74 C2H2-ZFPs—including 27 KRAB-ZFPs—show blastoderm-enriched expression, indicating their association with early embryo development. Overall, this study performs comprehensive annotation and expression profiling of C2H2 ZFPs in diverse chicken tissues, which gives new insights into the evolution and potential function of C2H2-ZFPs in avian species.

## 1. Introduction

Transcription factors (TFs) are DNA-binding proteins that act as key regulators of gene expression. In eukaryotes, Cys2His2 (C2H2)-type zinc finger proteins (C2H2-ZFPs) form the largest family of TFs [1,2]. C2H2-ZFPs rely on their 3′end zinc finger (ZF) arrays for DNA recognition, with one ZF routinely recognizing three nucleotides. However, unconventional recognition is also prevalent, as demonstrated by recent studies [3,4]. Increasing evidence suggests the crucial roles of C2H2-ZFPs in various biological processes, such as embryonic development, placentation, neurogenesis, immunity, and tumors [5,6,7,8,9,10,11]. Notably, a class of C2H2-ZFPs bearing N-terminal Krüppel-associated box (KRAB) domains (namely KRAB-ZFPs or KZFPs) has specifically evolved to silence transposable elements (TEs), which are mobile DNA elements abundant in diverse eukaryote genomes [12,13,14,15]. Relative to other TFs, C2H2-ZFPs are known to undergo fast evolution and expansion, as demonstrated by their divergence across species [16,17,18,19]. Nevertheless, most current studies on C2H2-ZFPs are constrained to mammals, while other eukaryotic lineages, such as birds, remain largely uncharacterized.

Among the over ten thousand avian species, the chicken (*Gallus gallus*) is particularly important as both economic animal for meats and a research model for birds. The first draft of the chicken genome was sequenced twenty years ago and has been further improved by later studies [20,21,22]. Many C2H2-ZFPs are reported to be shared between mammals and birds and tend to play conserved roles, such as YY1 for embryonic development [23], GATA4 and DNMT1 for sex determination [24,25], KLF proteins for adipogenesis and connective tissues [26,27,28], and CTCF for three-dimensional chromatin organization [29,30,31]. On the other hand, the chicken genome has remarkable differences relative to mammals regarding the occurrence and function of C2H2-ZFPs. For example, the chicken has a smaller number of KRAB-ZFPs, which are particularly abundant in mammalian genomes [32,33]. In addition, the chicken also lacks some other C2H2-ZFP genes, such as ZFP36 (also known as TTP), which has important antiviral function in mammals [34]. Despite these studies, the comprehensive characterization of C2H2-ZFPs in the chicken genome is still lacking, and the expression profiles of C2H2-ZFPs across chicken tissues also remain unclear.

In mammals, many C2H2-ZFPs have been previously reported to show distinct expression patterns across tissues, with genome-wide binding and function having been investigated for many of them before [13,33,35]. With the advances of high-throughput sequencing techniques, large amounts of data have accumulated for different chicken tissues. Impressively, the Functional Annotation of Animal Genomes (FAANG) project [36] was launched for the multi-omics profiling of various tissues in different economic animals, such as chicken, cow, and pig [36,37]. Notably, FAANG applied high-throughput techniques such as Chromatin Immunoprecipitation sequencing (ChIP-seq) and RNA sequencing (RNA-seq) to profile the epigenomic and transcriptomic data for dozens of chicken tissues [30,38,39,40]. Apart from the FAANG project, other researchers around the world have also performed transcriptomic profiling for a variety of chicken tissues [41,42,43,44]. Together, the huge amount of data generated by those studies enables the comprehensive characterization of C2H2-ZFP expression across chicken tissues.

This study aims to uncover the genomic distribution, evolutionary signature, and tissue-specific expression of C2H2-ZFPs in chicken. We performed comprehensive annotations of chicken C2H2-ZFPs and characterized their expression patterns in 32 tissues by using over 100 transcriptomic data. Our study provides novel insights into the evolution and expression of chicken C2H2-ZFPs and will facilitate functional studies of C2H2-ZFPs in chickens.

## 2. Results

### 2.1. Comprehensive Annotation of C2H2-ZFPs in Chicken Genome

**Figure 1:**
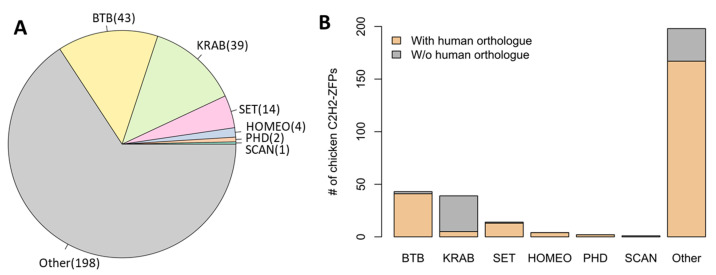
Statistics of the C2H2-ZFPs annotated in the chicken genome. (**A**) Pie plot shows the numbers of different groups of chicken C2H2-ZFPs. (**B**) Comparison of the different groups of chicken C2H2-ZFPs w/wo human orthologues.

We performed a genome-wide annotation of chicken C2H2-ZFPs by using a procedure similar to previous studies [14,45]. Apart from the C2H2-ZF array, several associated effector domains were also analyzed, including KRAB and BTB/POZ, which frequently occur in C2H2-ZFPs, and several other domains that occur with relatively low frequency [1,19]. In total, we annotated 301 putative C2H2-ZFPs in the chicken genome, with 39 and 43 of them bearing KRAB and BTB/POZ domains, respectively (Figure 1A, Table S1). Of note, KRAB-ZFPs make up 13.0% of all chicken C2H2-ZFPs, which is a much lower proportion relative to humans. A previous study estimated that about 58.2% (394/677) of human C2H2-ZFPs have a KRAB domain [18]. This is expected given the arms race between KRAB-ZFPs and TEs and the relatively low TE abundance in the chicken genome [15,33]. In addition, we annotated dozens of C2H2-ZFPs associated with other effector domains, including SET (n = 14), homeodomain (n = 4), PHD (n = 2), and SCAN (n = 1) (Figure 1A, Table S1).

Next, we assessed the divergence of the annotated C2H2-ZFPs between chicken and mammals. In brief, we retrieved the orthologue-matching information of chicken against humans and mice based on Ensembl orthology annotation [46], and then inspected whether each chicken C2H2-ZFP has a matched orthologue in mammals. Since the orthologue pairing results against humans and mice were quite similar (Table S1), we only used the human data for visualization. Overall, 77.1% (232/301) of the chicken C2H2-ZFPs have human orthologues, and the percentage differs remarkably across groups. Specifically, most C2H2-ZFPs with BTB (95.3%), SET (92.9%), HOMEO (100%), and PHD (100%) domains or those lacking the analyzed effector domains (84.3%) have human orthologues (Figure 1B). In contrast, only 12.8% of chicken KRAB-ZFPs have matched human orthologues (Figure 1B), suggesting their divergence between birds and mammals. We further compared these results against the zebra finch, another widely-studied bird species that is estimated to have split from chicken around 90 million years ago [47]. Surprisingly, most chicken KRAB-ZFPs (87.2%) also lack orthologues in zebra finch (Table S1), suggesting that KRAB-ZFPs are not conserved across different bird lineages. Of note, the single SCAN-ZFP annotated in this study also lacks human and mouse orthologue (Table S2). These data indicate that most KRAB-ZFPs and SCAN-ZFP in the chicken genome are lineage-specific, while other types of C2H2-ZFPs are usually shared by birds and mammals.

### 2.2. Dozens of Chicken C2H2-ZFPs Form Gene Clusters

**Figure 2:**
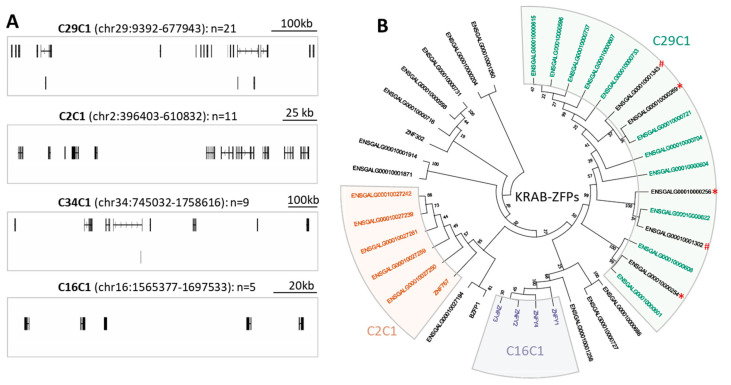
Representative C2H2-ZFP gene clusters identified in chicken genome. (**A**) This figure shows the four C2H2-ZFP gene clusters located in chromosome 29, 2, 34, and 16, respectively. These four clusters are termed as C29C1, C2C1, C34C1, and C16C1 based on their genomic distribution. The genomic coordinates and number of C2H2-ZFPs for each cluster are denoted. (**B**) Consensus tree of the 39 annotated KRAB-ZFPs was generated by using Maximum Likelihood (ML) method. The KRAB domains of these KRAB-ZFPs were used for phylogenetic analysis. The tree demonstrates that most KRAB-ZFPs for the three major gene clusters (C29C1, C2C1, and C16C1) form their own clades, as highlighted by color. The numbers on the tree represent the bootstrap scores. The three KRAB-ZFPs from scaffold JAENSK010000383 (denoted by “*”) and two KRAB-ZFPs from scaffold JAENSK010000384 (denoted by “#”) cluster closely with the 11 KRAB-ZFPs from C29C1.

In mammals, numerous C2H2-ZFPs, particularly those belonging to KRAB-ZFPs, tend to form gene clusters through gene duplication [12,13]. Here, we inspected the occurrence of C2H2-ZFP clusters in the chicken genome by following previous studies [16,45]. In total, we identified 12 gene clusters—each contains at least three C2H2-ZFPs (Table S2), with a total of 73 C2H2-ZFPs (including 33 KRAB-ZFPs) residing in them. Among these clusters, four have at least five C2H2-ZFPs (Figure 2A), which are located at chromosome 2, 16, 29, and 34, respectively. Impressively, the largest cluster (termed C29C1) is located at chr29 and contains 21 C2H2-ZFPs, including 16 that belong to KRAB-ZFPs (Figure 2A, Table S2). None of the C2H2-ZFPs in C29C1 have human orthologues (Table S2), suggesting their lineage-specificity. We further inspected the other three clusters (termed as C2C1, C16C1, and C34C1) and found that C2C1 and C16C1 also mainly consist of KRAB-ZFPs, while C34C1 is mainly made up of C2H2-ZFPs that lack the analyzed effector domains (Figure 2A, Table S2).

Since KRAB-ZFPs frequently form gene clusters, we further determined their evolutionary relationship. We constructed a phylogenetic tree for the 39 annotated KRAB-ZFPs based on their KRAB domains (Figure 2B). We found that the KRAB-ZFPs from C29C1, C2C1, and C16C1 all tend to form their own clades (Figure 2B). For example, 11 of the 16 KRAB-ZFPs from C29C1 occur in the same clade. Of note, the five remaining KRAB-ZFPs in the same clade are from two unassigned scaffolds (JAENSK010000383 and JAENSK010000384) (Figure 2B, Table S2), indicating the C29C1 cluster may contain additional C2H2-ZFPs which are currently unassigned to chr29 due to the incomplete genome assembly. For the other two gene clusters C2C1 and C16C1, all the involved KRAB-ZFPs also form their own clades (Figure 2B). Together, these data suggest that dozens of C2H2-ZFPs form gene clusters in the chicken genome, and they are usually formed by duplicated KRAB-ZFPs.

### 2.3. The Chicken SCAN-ZFP Is Lineage-Specific and Has Tissue-Specific Expression

**Figure 3:**
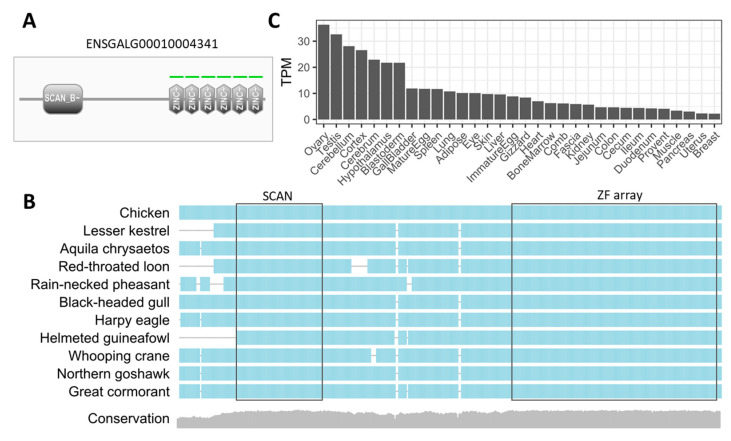
Characterization of the single SCAN-ZFP annotated in the chicken genome. (**A**) The domain structure of the annotated SCAN-ZFP according to the results from ScanProsite. The occurrence of the KRAB domain and ZF array is illustrated. (**B**) Sequence alignment profile of the chicken SCAN-ZFP and representative orthologues from ten other bird species. The sequences of the orthologous proteins were obtained by BLAST search against the NCBI nr database. Only the positions with no gaps in the chicken sequence are included for visualization. The conservation score, calculated by using Jensen–Shannon divergence, is plotted alongside the alignment. (**C**) Expression pattern of the annotated SCAN-ZFP in different chicken tissues. This figure was generated based on the normalized TPM values calculated from RNA-seq data.

Despite the fact that dozens of SCAN-ZFPs (including many with both SCAN and KRAB domains) exist in mammalian genomes [33,48], previous studies found no SCAN-ZFP in the chicken genome [16,17,18]. Surprisingly, we predicted a single SCAN-ZFP (Ensembl Gene ID: ENSGALG00010004341) which was missed by previous studies. It is located at chr31:1311105-1316631 and contains a SCAN domain and a C2H2-ZF array (Table S1). The presence of the SCAN domain at the N-terminal and C2H2-ZF array at C-terminal (Figure 3A) was further confirmed by using ScanProsite [49]. But why was this gene not identified by previous studies? We searched its sequence against different assemblies of chicken genome by using UCSC blat [50], and found that its sequence could be aligned in full-length only after GalGal5 was released in 2015. In contrast, earlier assemblies, such as GalGal3 and GalGal4, both lack the SCAN region. Therefore, we speculate that this gene was missed in previous studies due to the incompleteness of earlier genome assemblies.

Next, we examined if this SCAN-ZFP has orthologues in other species, such as mammals and other bird species. According to Ensembl orthology annotation, this gene has an orthologue in zebra finch yet lacks an orthologue in humans or mice (Table S1). We further searched this gene against the NCBI nr database and found that it has highly similar orthologue in many avian species, such as *Phasianus colchicus* (ring-necked pheasant), *Numida meleagris* (helmeted guineafowl), and *Accipiter gentilis* (Northern goshawk). We then performed multiple sequence alignment for the chicken SCAN-ZFP and ten representative homologous sequences from different bird species. The result confirmed that both the SCAN and C2H2-ZF domains exist in all the inspected proteins, and their corresponding loci show relatively high sequence conservation (Figure 3B). These results indicate the high conservation of this SCAN-ZFP across bird species.

After confirming the conservation of the identified SCAN-ZFP in birds, we wondered if this gene is expressed in specific tissues. For this purpose, we collected 101 RNA-seq datasets from public resources, which cover 32 different chicken tissues or organs (Table S3). Through the comparison of the normalized expression levels, we found this gene has a highly tissue-specific expression pattern. Particularly, this gene is most highly expressed in the two reproductive tissues—ovary and testis—with Transcript per Million (TPM) values of 36.3 and 32.6 respectively (Figure 3C), which is notably high since most TFs only have low or moderate expression. It is also highly expressed in central nervous system (CNS) and blastoderm, yet is only moderate or weakly expressed in all other inspected tissues. For example, it is almost unexpressed in uterus and breast, with TPM values of 2.3 and 2.2, respectively (Figure 3C). Together, these data suggest the existence of a SCAN-ZFP in chicken genome and many other bird species, and indicate that this gene may be important for reproduction, early development, and CNS, as implied by its high expression in related tissues.

### 2.4. Global Expression Profile of C2H2-ZFPs across Chicken Tissues

By integrating the 101 RNA-seq datasets collected from multiple previous studies (Table S3), we inspected the expression pattern of the annotated chicken C2H2-ZFPs across 32 different tissues and organs. We first performed hierarchical clustering by using the normalized expression matrix (Table S4), and confirmed that the clustering pattern of these samples agrees well with the relationship between the corresponding tissues and organs (Figure S1). For example, samples from the immune systems (i.e., spleen and bone marrow), digestion systems (i.e., cecum, colon, duodenum, ileum, provent), and CNS (i.e., cerebellum, cortex, hypothalamus) each form their own clades (Figure S1). These results confirmed the reliability of the collected transcriptomic data. Subsequently, we used the normalized expression matrix for comparative analysis.

**Figure 4:**
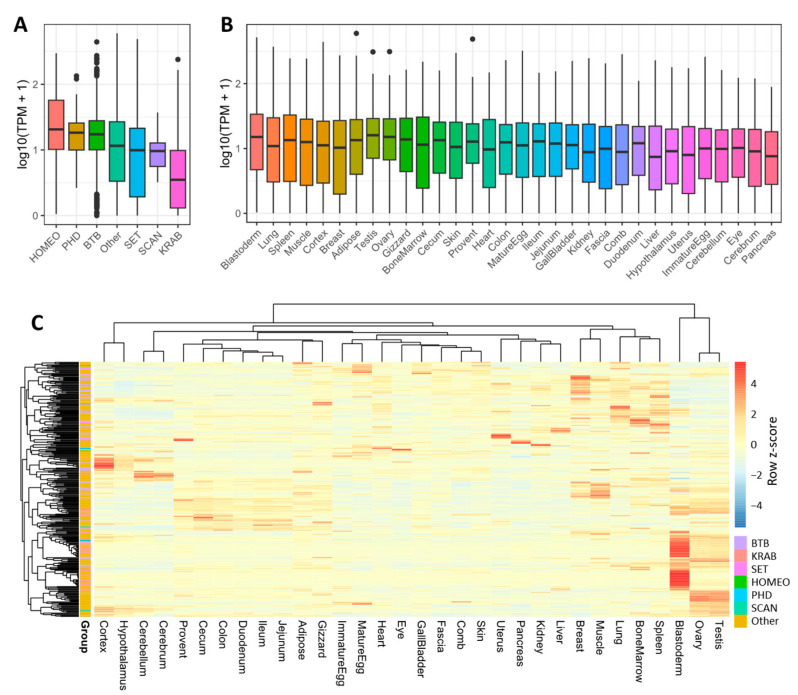
Global expression patterns of different groups of C2H2-ZFPs across chicken tissues. (**A**) Comparison of C2H2-ZFPs expression across different groups. The boxplot is arranged by the averaged TPM values. (**B**) Comparison of C2H2-ZFP expression across different chicken tissues. The boxplot is arranged by the averaged TPM values. (**C**) Heatmap showing the expression profile of C2H2-ZFPs across 32 different chicken tissues. This heatmap was produced based on the normalized TPM values. The color gradients represent the row z-score.

We first compared the expression of different groups of C2H2-ZFPs (Figure 4A). Overall, KRAB-ZFPs showed the lowest expression level, while BTB-ZFPs showed much higher expression (Figure 4A). Next, we compared the expression levels across different samples, and found that the blastoderm shows the highest levels of C2H2-ZFP expression (Figure 4B). Interestingly, previous studies suggest important roles of many ZFPs during early mammalian embryo development [9,10,12,51,52]. This indicates that ZFPs may also play particularly important roles in the early development of chickens. Lastly, we compared the expression of each C2H2-ZFP in the 32 analyzed tissues and organs. The data clearly demonstrate that many C2H2-ZFPs have tissue-specific expression (Figure 4C). In particular, many genes show enriched expression in blastoderm, consistent with the relatively high expression observed in this tissue. Together, these data suggest the high tissue-specificity of C2H2-ZFP expression across chicken tissues.

### 2.5. Numerous Chicken C2H2-ZFPs Are Specifically Expressed in Blastoderm

**Figure 5:**
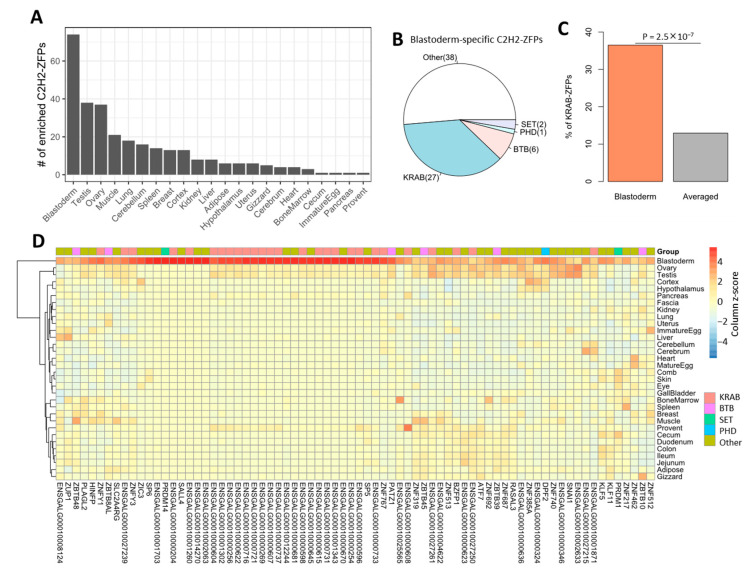
Characterization of the C2H2-ZFPs with tissue-specific expression patterns. (**A**) The numbers of tissue-specific C2H2-ZFPs identified for each chicken tissue. (**B**) The numbers of different groups of C2H2-ZFPs with enriched expression in blastoderm. (**C**) Comparison of the proportions of KRAB-ZFPs in blastoderm-enriched C2H2-ZFPs relative to all C2H2-ZFPs. The *p*-value calculated by using the Binomial test is indicated. (**D**) The cross-tissue expression profiles for the 74 blastoderm-enriched C2H2-ZFPs. This heatmap was produced based on the normalized TPM values. The color gradients represent the column z-score.

After revealing the global expression patterns of chicken C2H2-ZFPs, we further performed quantitative analysis to identify those with significantly enriched expression in each tissue. Of the C2H2-ZFPs focused on in our study, 65.1% (196 out of 301) of them show enriched expression in one or more tissues (Figure S2), suggesting that most C2H2-ZFPs have tissue-specific expression. These genes are hereby denoted as tissue-specific C2H2-ZFPs. We further demonstrate that the numbers of tissue-specific C2H2-ZFPs differ remarkably across tissues, with blastoderm—which has 74 enriched C2H2-ZFPs—ranking 1st (Figure 5A, Table S5). In addition, testis and ovary each also have almost 40 enriched C2H2-ZFPs and are ranked as the 2nd and 3rd, respectively (Figure 5A, Table S5). Of note, the higher number of tissue-specific C2H2-ZFPs in these three tissues is consistent with the results from the global expression profile (Figure 4 and Figure S2).

Given that large numbers of C2H2-ZFPs are highly expressed in blastoderm, we inspected whether they belong to be specific types of C2H2-ZFPs. Of the 74 blastoderm-enriched C2H2-ZFPs, the majority are KRAB-ZFPs (n = 27) or genes lacking the analyzed effector domains (n = 38). In contrast, only 10 blastoderm-enriched C2H2-ZFPs (6 for BTB, 1 for PHD, 2 for SET) do not belong to KRAB-ZFPs (Figure 5B). Given that there are only 39 KRAB-ZFPs in total, these data suggest that KRAB-ZFPs are associated with blastoderm at a significantly higher frequency than expected (Figure 5C). Of note, most of the C2H2-ZFPs from the largest annotated gene cluster (C29C1) also show enriched expression in blastoderm (Figure 5D, Table S5). Interestingly, we previously found that many KRAB-ZFPs are also highly expressed in mouse embryonic stem cells [13], indicating that KRAB-ZFPs are associated with early development in both mammals and birds. Together, these data suggest that high proportions of chicken C2H2-ZFPs show tissue-specific expression, and many C2H2-ZFPs (particularly KRAB-ZFPs) are highly expressed in blastoderm, implying their association with early embryo development.

## 3. Discussion

As the largest class of eukaryotic TFs, C2H2-ZFPs have been extensively studied in mammals yet remain poorly characterized in birds. Here, we performed a comprehensive study of C2H2-ZFPs in chicken. We annotated a total of 301 C2H2-ZFPs in the chicken genome, with 103 associated with several effector domains, particularly KRAB and BTB. In mammals, it has long been recognized that KRAB-ZFPs have been greatly expanded for TE repression [13,15,45,48]. However, we only annotated 39 KRAB-ZFPs in the chicken genome, which is much less than the ~400 KRAB-ZFPs in the human genome [33]. This is likely due to the low abundance of TEs in the chicken genome relative to mammals. Despite the relatively small number of chicken KRAB-ZFPs, we found that they usually lack orthologues in mammals or even other bird species (i.e., zebra finch), indicating that KRAB-ZFPs are undergoing fast evolution in birds. In addition, KRAB-ZFPs form several gene clusters in the chicken genome. In particular, we noticed that the largest gene cluster at chr29 (C29C1) contains 16 KRAB-ZFPs. Given that five other KRAB-ZFPs from unassigned genome scaffolds are closely related to C29C1 according to phylogenetic analysis, we suspect the C29C1 cluster may contain as many as 21 KRAB-ZFPs. Transcriptomic analysis suggests that most C2H2-ZFPs from C29C1 are specifically expressed in the blastoderm. Then, does this KRAB-ZFP gene cluster play a role during the chicken embryo development? Previously, we successfully applied CRISPR/Cas9 engineering to delete multiple KRAB-ZFP clusters in mice to study their function [13]. We expect that a similar strategy may also be applied to investigate the function of chicken KRAB-ZFP clusters.

The SCAN domain, which is derived from a Gypsy/Ty3-like retrotransposon, frequently occurs at the N-termini of C2H2-ZFPs in mammals [53,54]. Despite the fact that about fifty SCAN-containing C2H2-ZFPs (including ~30 containing both SCAN and KRAB domains) are found in human genome [33,48,55], previous studies failed to identify any SCAN-containing C2H2-ZFP in chicken genome [16,17,18]. Surprisingly, our study uncovered a SCAN-ZFP in the chicken genome. To reconcile our and previous studies, we found that this SCAN-ZFP was missed by previous studies due to the incompleteness of earlier chicken genome assemblies. Interestingly, this SCAN-ZFP is likely conserved across birds yet absent in mammals, given that: (1) BLAST searching of this gene against the NCBI nr database identified highly conserved orthologous genes in dozens of avian species but not mammals. (2) The Ensembl orthology pairing data suggest that this gene has an orthologue in zebra finch but not human or mouse. However, we are still unclear if this gene newly emerged in birds, or whether it has an ancient origin that specifically was lost in mammals. Taking advantage of public transcriptomic data, we further demonstrate that this SCAN-ZFP has a highly tissue-specific expression profile, with its expression being most abundant in ovary, testis, brain, and blastoderm. These data indicate that this gene may regulate reproduction, CNS, and early embryo development. As the only chicken SCAN-ZFP reported so far, it is highly desirable to further investigate its biological role in these associated tissues.

Apart from the genomic annotation of chicken C2H2-ZFPs, we also determined their expression signatures by integrating over 100 RNA-seq datasets for 32 chicken tissues. The overall expression abundance differs across different groups of C2H2-ZFPs, with KRAB-ZFPs showing the lowest expression level, while HOMEO- and PHD-ZFPs show relatively high expression level. Of note, previously we found that KRAB-ZFPs also exhibit relatively low expression abundance across mouse tissues [13]. Cross-tissue comparison suggests that most chicken C2H2-ZFPs show a tissue-specific expression pattern, with 65.1% of them showing significantly enriched expression in specific tissues. Of the 32 analyzed tissues, the blastoderm appears to be unique, given that many C2H2-ZFPs are specifically expressed there. Specifically, 74 C2H2-ZFPs, including 27 KRAB-ZFPs, show blastoderm-specific expression, indicating their potential involvement in early embryo development. Apart from the blastoderm, the testis and ovary each also shows enriched expression of nearly 40 C2H2-ZFPs. Many C2H2-ZFPs have been previously reported to play important roles during early mammalian embryo development [9,10,12,51,52]. However, the exact roles of C2H2-ZFPs during the early embryo development and reproduction in chickens remain to be investigated.

In summary, this study represents a comprehensive analysis of the occurrence, evolution, and expression profiles of C2H2-ZFPs in the chicken. Our study improves our understanding of the evolution and potential function of C2H2-ZFPs in avian species, and the tissue-specific C2H2-ZFPs identified in this study may serve as promising candidates for further functional studies.

## 4. Materials and Methods

### 4.1. Genome-Wide Annotation of Chicken C2H2-ZFPs

We screened the putative C2H2-ZFPs across the chicken genome by using a procedure modified from previous studies [14,45]. In brief, we retrieved the protein sequences for the chicken (GRCg7b) from the Ensembl database release 106 [46], and then used the *hmmsearch* function of HMMER v3.4 [56] to scan for the presence of interested protein domains, with settings: -noali -E 0.05 -incE 0.05. In addition to the C2H2 zinc finger (ZF-C2H2, PF00096), we also analyzed several related domains including BTB/POZ (PF00651), Homeodomain (PF00046), KRAB (PF01352), PHD (PF00628), SCAN (PF02023), and SET (PF00856). The hmm profiles for these domains were downloaded from the InterPro database [57].

When a predicted C2H2-ZFP had multiple isoforms, a canonical isoform was selected step-by-step following these criteria: (1) When an effector domain (e.g., KRAB and BTB) was present in some but not all isoforms, only the isoforms containing the effector domain were retained; (2) For the isoforms showing different numbers of ZFs, the one with the largest number is retained; (3) When multiple isoforms had the same effector domain and ZF arrays, the longest one was selected. The statistics for the canonical isoform of each predicted chicken C2H2-ZFP are summarized in Table S1.

### 4.2. C2H2-ZFP Cluster Analysis

The C2H2-ZFP clusters were detected by using a procedure similar to previous studies [16,45]. In brief, the genomic coordinates for each predicted C2H2-ZFP were retrieved from Ensembl by using BioMart [46]. After that, we used BEDtools [58] to merge any C2H2-ZFP genes that occurred less than 250 kb from each other to form gene blocks. Lastly, we counted the number of C2H2-ZFPs in each gene block, and only kept those with at least 3 C2H2-ZFPs for further analyses.

### 4.3. C2H2-ZFP Orthologue Analysis

To determine if each chicken C2H2-ZFP bore any orthologues in mammals, we retrieved the orthologue information of the chicken relative to human and mouse by using the BioMart from Ensembl [46]. The orthologous genes across species were annotated by considering both the gene order conservation and whole genome alignment. More details are available at the Ensembl website: https://mart.ensembl.org/info/genome/compara/Ortholog_qc_manual.html (accessed on 20 September 2024) The orthologue table was then used to evaluate the conservation of predicted chicken C2H2-ZFPs in mammals.

### 4.4. BLAST Searching

To obtain the putative orthologous genes of the chicken SCAN-ZFP in other species, we performed online search of the annotated chicken SCAN-ZFP protein sequence against the NCBI nr database by using BLASTP [59] with default settings. Then, the protein sequences for the top ranked homologous genes from ten different bird species (each species having a matched gene) were retrieved, and then these proteins, together with the chicken SCAN-ZFP, were subjected to multiple sequence alignment and conservation evaluation.

### 4.5. Sequence Conservation Evaluation

We evaluated the sequence conservation for each amino acid of the annotated chicken SCAN-ZFP protein relative to other putative orthologous proteins from different bird species. We first performed multiple sequence alignment together with putative orthologous genes in other bird species by using ClustalW v2.1 [60]. Subsequently, the Jensen–Shannon divergence for each position was calculated following a previous study [61], and then this was used for visualization.

### 4.6. Phylogenetic Tree Construction

We performed phylogenetic analysis for the annotated KRAB-ZFPs by using the protein sequences of their KRAB domains. The KRAB sequences were extracted based on the coordinates from the hmmsearch results and were aligned with Clustalw v2.1 [60]. Finally, the ML tree was constructed using the JTT model with MEGA X [62]. The consensus tree was inferred from 1000 bootstrap replicates.

### 4.7. RNA-Seq Analysis

Raw reads were trimmed with Trim Galore! v0.6.4 [63], and then aligned to the chicken reference genome (GRCg7b) by using STAR v2.7.3a [64]. Gene-level read counts were calculated by using the featureCount function from subread v2.0.0 [65]. Differentially expressed genes were identified by using DESeq2 [66], with settings: FDR < 0.05, |log2fold| > 1. To identify the genes with enriched expression in a specific tissue, we performed differential expression analysis of this tissue against all other tissues analyzed in this study. We performed this analysis repeatedly for each tissue to identify all tissue-specific genes.

TPM values for each gene were calculated with RSEM v1.3.2 [67] with default settings, and then merged together into an expression matrix. The merged matrix was filtered to discard genes with TPM value less than 1 in all samples or above 10,000 in any samples, and further normalized by using the Trimmed mean of M values (TMM) method by using the normalize_matrix function from ExprX v0.0.3 [68]. The normalized expression matrix was used for subsequent comparison across chicken tissues.

### 4.8. Statistical Analysis and Data Visualization

All statistical analyses were performed with the R statistical programming language [69]. Heatmaps for gene expression clustering were generated using pheatmap [70].

### 4.9. Data Availability

The RNA-seq data used in this study were collected from the FAANG project (Accession: PRJEB26695, PRJEB53920) and multiple other studies [39,40,41,42]. They include 101 datasets for 32 chicken tissues or organs. The sources of these data are summarized in Table S3.

## Data Availability

The data analyzed during the current study are collected from public studies, and are summarized in Table S3.

## Supplementary Materials

The following supporting information can be downloaded at: https://www.mdpi.com/article/10.3390/ijms251910525/s1.
