# Supplementary material for: Comprehensive Annotation and Expression Profiling of C2H2 Zinc Finger Transcription Factors across Chicken Tissues
Source: Int J Mol Sci. 2024 Sep 30;25(19):10525. doi: 10.3390/ijms251910525 (PMC11476951; doi:10.3390/ijms251910525)
Supplement: Supplementary file 1 [file ijms-25-10525-s001.zip › Supplemental Figure - 0824.pptx]

## Slide 1
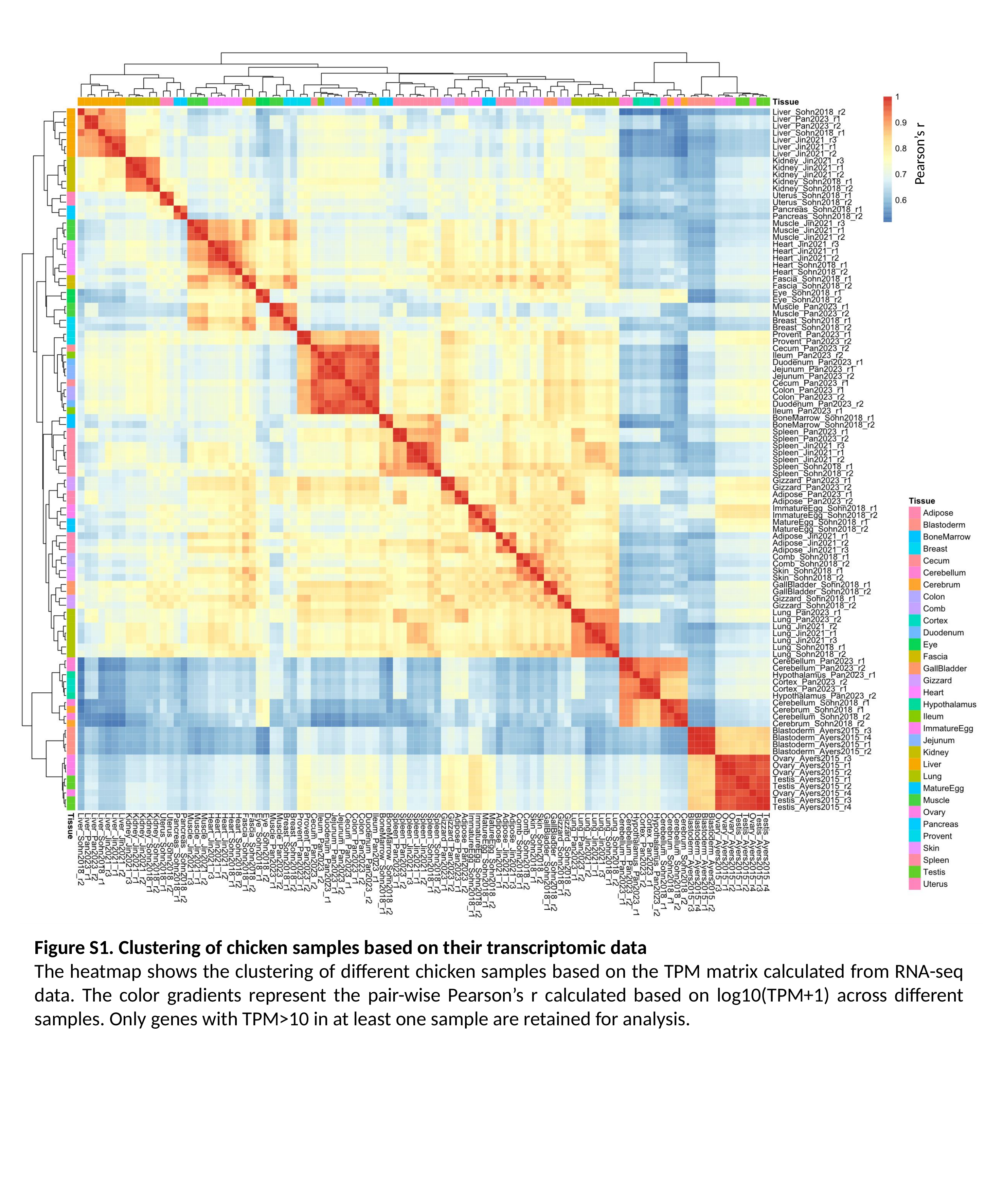

Pearson’s r
Figure S1. Clustering of chicken samples based on their transcriptomic data
The heatmap shows the clustering of different chicken samples based on the TPM matrix calculated from RNA-seq data. The color gradients represent the pair-wise Pearson’s r calculated based on log10(TPM+1) across different samples. Only genes with TPM>10 in at least one sample are retained for analysis.

## Slide 2
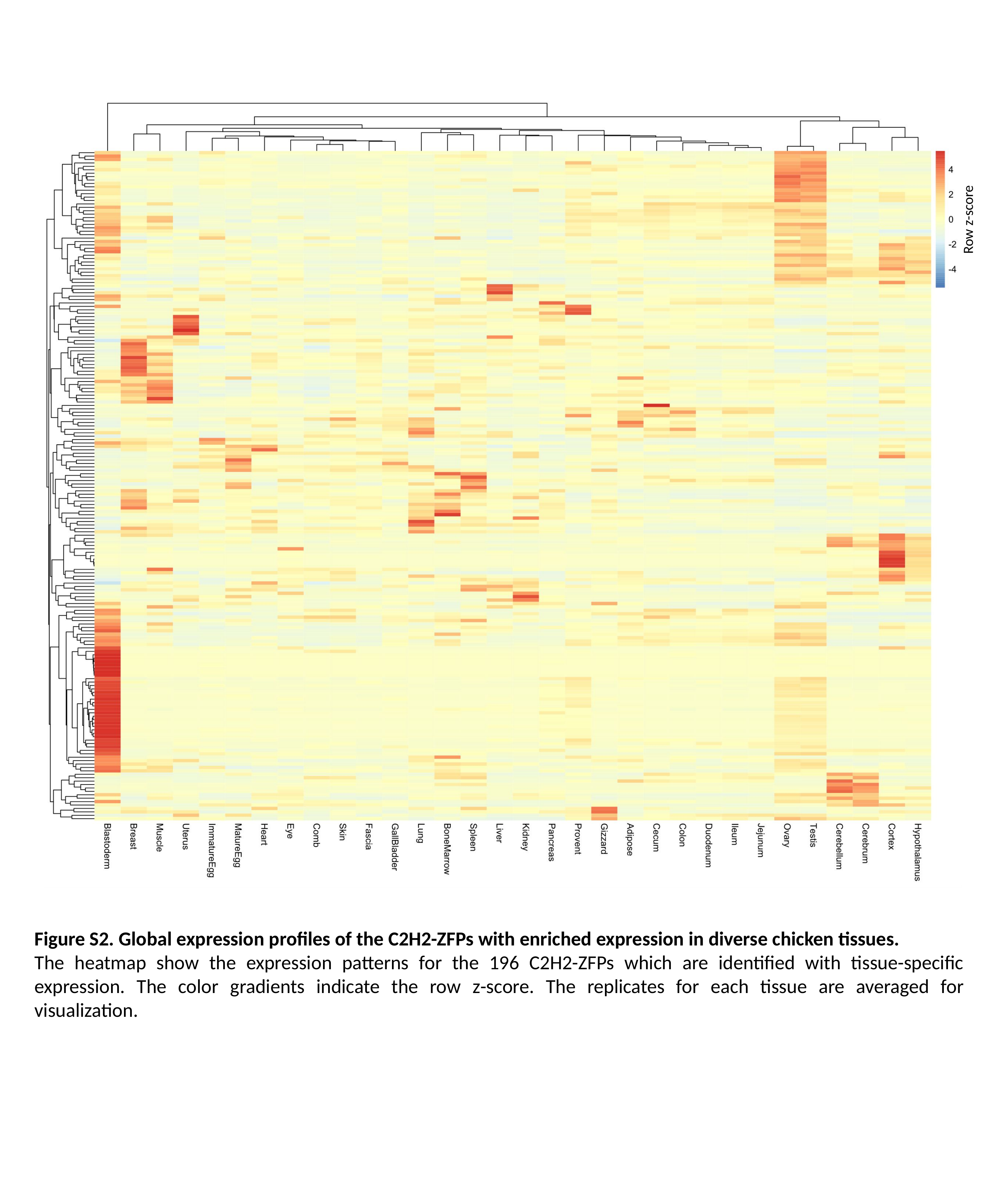

Row z-score
Figure S2. Global expression profiles of the C2H2-ZFPs with enriched expression in diverse chicken tissues.
The heatmap show the expression patterns for the 196 C2H2-ZFPs which are identified with tissue-specific expression. The color gradients indicate the row z-score. The replicates for each tissue are averaged for visualization.
